# Loss of FAM134B increased endoplasmic reticulum stress and induced cell autophagy in breast cancer

**DOI:** 10.3389/fimmu.2025.1652888

**Published:** 2025-11-10

**Authors:** Zhaoqi Zhang, Yan Fang, Zhibing Qiu, Shuning Ding, Deyue Liu, Wanqiu Huang, Li Zhu

**Affiliations:** 1 Department of Breast and Thyroid Surgery, Shanghai General Hospital, School of Medicine, Shanghai Jiao Tong University, Shanghai, China; 2 Department of Digestive Disease, Huashan Hospital, Fudan University, Shanghai, China; 3 Key Laboratory of Systems Biomedicine (Ministry of Education), Shanghai Centre for Systems Biomedicine, Shanghai Jiao Tong University, Shanghai, China

**Keywords:** breast cancer, FAM134B, endoplasmic reticulum stress, autophagy, apoptosis

## Abstract

**Objective:**

Breast cancer is a leading cause of cancer-related mortality, and the most prevalent malignant neoplasm amongst women worldwide. This study aimed to explore the role of FAM134B in breast cancer progression.

**Methods:**

The correlation between FAM134B expression and the prognosis of breast cancer patient was analyzed using the Kaplan-Meier Plotter database. qRT-PCR was used to quantify *FAM134B* mRNA level, whereas western blotting was employed to detect th expression of FAM134B, autophagy-associated proteins, and endoplasmic reticulum (ER) stress related proteins. Cell proliferation was assessed via CCK-8 and colony formation assays. Cell apoptosis rate was measured by flow cytometry. Autophagosomes formation was observed under a transmission electron microscopy, and the expression of LC3 protein in cells was detected by immunofluorescence. The *in vivo* function of FAM134B was verified using a tumor xenograft model in nude mice.

**Results:**

High expression of FAM134B in breast cancer patients was correlated with reduced overall survival and disease-free survival. Both FAM134B mRNA and protein levels were significantly higher in breast cancer cells than normal breast epithelial cells. Downregulation of FAM134B suppressed the proliferation of breast cancer cells and increased their apoptosis rates. Furthermore, silencing FAM134B triggered autophagy and ER stress in breast cancer. In nude mice, FAM134B knockdown also inhibited breast cancer progression and induced autophagy.

**Conclusion:**

Downregulation of FAM134B inhibited the development of breast cancer through inducing apoptosis, autophagy, and ER stress of breast cancer cells.

## 1 Introduction

Breast cancer is the most common malignancy among women globally, with approximately 2.3 million new diagnoses in 2020, surpassing all other cancers in 159 out of 185 countries and regions worldwide (1). According to the data of American Cancer Society, the projected breast cancer incidences and fatalities in women, within the USA, by the year 2024, amount to 32% and 21%, respectively (2). With higher rates prevailing in nations with advanced human development indices, this might be attributed not only to augmented frequencies of potential etiologic factors such as estrogen supplementation, contraceptive medications, high-calorie, low-fiber diets, alcohol intake, obesity, delayed childbearing or amenorrhea (3, 4). To reduce the disease burden of breast cancer, it is crucial to investigate the underlying mechanisms of carcinogenesis and identify potential therapeutic strategies.

The endoplasmic reticulum (ER) is a multifaceted organelle that possesses a pivotal function in protein synthesis, modifications, and transport (5). The intrinsic protein-folding capacity of the ER is exceptionally vulnerable to genetic and environmental stress, causing accumulation of misfolded or unfolded proteins within the ER lumen, a condition known as ER stress (6). Prolonged ER stress can trigger cellular self-destruction mechanisms. ER-phagy (reticulophagy) is a specialized form of selective autophagy, in which fragments of the ER are engulfed by autophagosomes via specific receptors and subsequently delivered to lysosomal degradation. This process helps restore cellular energy homeostasis and ER function (7, 8). It is widely acknowledged that ER stress enables cancer cells to adapt to the tumor microenvironment, thereby regulating cancer proliferation (9). Concurrently, ER stress can trigger apoptosis of cancer cells by activating p53 (10).

Family with sequence similarity 134-member B (FAM134B) was initially identified in esophageal squamous cell cancer (11). his gene encodes a 497-amino acid transmembrane protein localized to the cis-Golgi and ER membranes. FAM134B regulates ER turnover through targeted autophagy, thereby influencing intracellular ER stress levels (12). Furthermore, it has been reported that FAM134B regulated ER turnover by selective autophagy (13). In previous research, we have proved that FAM134B promotes epithelial-to-mesenchymal transition by Akt signaling in hepatocellular carcinoma (14). Nevertheless, the biological function and molecular mechanism of FAM134B in breast cancer remain unclear.

As a result, this research aims to investigate the role of FAM134B in breast cancer. We discovered that high FAM134B expression correlates with poor prognosis in breast cancer patients. Downregulation of FAM134B inhibited the development of breast cancer through regulating proliferation, apoptosis, autophagy and ER stress of breast cancer cells, which offers a novel insight and a potential therapeutic target for breast cancer.

## 2 Materials and methods

### 2.1 Bioinformatics analysis

The data of breast cancer patients were downloaded from the TCGA database (https://portal.gdc.cancer.gov). RNA-seq data from the TCGA-BRCA project was subjected to STAR workflow and extracted in transcripts per million (TPM) format to obtain gene expression and clinical data. Data processing and visualization were performed using R software (version 4.2.1). The prognostic value of FAM134B in breast cancer was analyzed through Kaplan-Meier Plotter database (https://kmplot.com/analysis/), including overall survival (OS) and disease-free survival (DFS).

### 2.2 Cell culture

The human normal breast epithelial cell line MCF-10A and human breast cancer cell lines MCF-7 and MDA-MB-231 were bought from Cobioer (Nanjing, China). MCF-10A cells were seeded in BEGM medium (BeNa Culture Collection, China), MCF-7 cells were cultured in RPMI-1640 medium (GIBCO, USA) and MDA-MB-231 cells were cultured using L15 medium (GIBCO). The conditioned medium contains 10% fetal bovine serum (FBS) and 100 U/mL penicillin-streptomycin (Sigma-Aldrich, USA). All of the cell lines used in current study have been tested to confirm the absence of mycoplasma contamination.

### 2.3 Cell transfection

Recombinant lentiviral vectors specifically designed for FAM134B (shFAM134B) and control non-targeting vector (shNC) were constructed by GenePharma (Shanghai, China). MCF-7 and MDA-MB-231 cells were transfected with the vectors at multiplicities of infection (MOI) ranging from 10 and 100, in the presence of 5 mg/mL polybrene. After transfection, the supernatant containing the viral particles was replaced with fresh DMEM. Following 72 h of incubation, the cells were selected with 2 μg/mL puromycin to establish stably transfected shFAM134B cell line.

### 2.4 Quantitative real time polymerase chain reaction

For qRT-PCR, total RNA was extracted from cells with TRIzol reagent (Invitrogen, USA) according to the manufacturer’s protocol. Complementary DNA (cDNA) was synthesized from RNA with Reverse Transcription Kit (Takara, Japan). Then qRT-PCR assay was performed by SYBR^®^ Premix Ex TaqTM (Takara) according to the instruction. The GAPDH was used as an endogenous control gene for normalizing the expression of *FAM134B*. The primer sequences are shown in **Table 1**.

**Table 1:** Primer sequences.

Gene	Sequences of primers (5'-3')
FAM134B forward	GACAGCATCACAGTTTCAGGGAGA
FAM134B reverse	AGACAGCCCATCCGTCTCCTT
GAPDH forward	TGGCCTTCCGTGTTCCTAC
GAPDH reverse	GAGTTGCTGTTGAAGTCGCA

### 2.5 Western blot

The proteins were extracted by radio immunoprecipitation assay (RIPA) buffer (Sigma-Aldrich, USA) and quantified by BCA Assay Kit (Sigma-Aldrich). A total of 20 μg protein was treated with 10% sodium dodecyl sulfate polyacrylamide gel electrophoresis (SDS-PAGE), and then transferred onto polyvinylidene fluoride (PVDF) membranes (Invitrogen). The membranes were blocked by 3% bovine serum albumin (BSA, Sigma-Aldrich), then primary antibodies including FAM134B, LC3B, IRE1α, p-IRE1α, PERK, p-PERK, CHOP and GAPDH were added and incubated overnight at 4°C. After that, secondary antibody horseradish peroxidase (HRP)-conjugated IgG (Abcam) was appended and incubated for 1 h at 37°C. The intensity of immunoreactive bands was normalized to that of GAPDH by ImageJ 1.4 software.

### 2.6 Proliferation assay

Cell viability was detected by cell counting kit-8 (CCK-8, Yeasen, China). Breast cancer cells were seeded into a 96-well plate at a density of 3 × 10^3^ cells/ml after transfection. The culture medium was added to 10 μl CCK-8 solution and cultured for 1.5 h, and the optical density (OD) value was measured at 450 nm at 0, 24, 48 and 72 h.

### 2.7 Colony formation assay

The breast cancer cells were cultured in a 6‐well plate (1×10^3^ cells per well) and incubated for 1 week at 37°C. Cells were then washed twice with PBS, fixed with 4% formaldehyde for 15 min, and stained for 30 min with Giemsa staining solution (Beyotime Biotechnology, China). Colonies with a diameter ≥ 100 μm were counted, and the assay was performed in triplicate.

### 2.8 Flow cytometry assay

The apoptotic rate of cells was determined under the guidelines of Annexin V-FITC/PI Apoptosis Detection Kit (KeyGEN Biotech, China). MCF-7 and MDA-MB-231 cells were sequentially stained with Annexin V-FITC and propidium iodide (PI), followed by incubation in a dark environment for 15 min. Subsequently, the status of cell apoptosis was calculated using a MoFlow flow cytometer (Beckman Coulter, USA).

### 2.9 Transmission electron microscopy

The cells were cultured in 6-well plates, gently detached by 0.25% trypsin, and fixed with 4% glutaraldehyde at 4°C for 2 h. Cells were then fixed with 1% osmium tetroxide for another two hours, stained with filtered uranyl acetate, and dehydrated using ethanol and acetone. Subsequently, the cells were encapsulated in Epon resin, and both semi-thin (0.5 μm) and ultra-thin (60 nm) sections were meticulously prepared. Sections were stained with lead citrate and uranyl acetate, then observed under a Hitachi H-7500 transmission electron microscope (Hitachi, Japan), and images were captured using a sophisticated Gatan780 system.

### 2.10 Immunofluorescence microscopy experiment

Cells were rinsed thrice with PBS and fixed with freshly prepared methyl alcohol at 20°C for 20 min. Cells were exposed to primary antibody (Abcam) overnight at 4°C, and subsequent to washing by PBS, stained with fluorescent secondary antibody (Abcam) for 1 h at room temperature. Post rinsing with PBS, cells were stained with 4,6-diamidino-2-phenylindole (DAPI) for 15 min, and washed thrice with PBS. Cell images were acquired using a confocal microscope (Nikon, Japan).

### 2.11 Tumor xenograft *in vivo*

The study was approved by the Institutional Animal Care and Use Committee of Shanghai General Hospital, School of Medicine, Shanghai Jiao Tong University (Approval No. 202501001). All animals received humane care according to the criteria outlined in the Guide for the Care and Use of Laboratory Animals. The mice were euthanized if they showed any adverse signs or symptoms of diseases which were beyond our intention in the experimental protocol. A total of 18 BALB/c nude mice (female, 5–6 weeks old, 18–22 g weight, achieved from the Laboratory Animal Center of Shanghai Jiao Tong University) were randomly divided into 6 groups (3 mice each group). Mice were kept in the animal house facility under a 12-h reversed light/dark cycle, 22°C with free access to water and food. Next, 0.2 mL of above cell suspension that contained 2 × 10^4^ cells was injected into the back of each mouse. The experiment lasted three weeks, and the mice in each group were monitored twice a week to the end of the experiment. After 3 weeks, mice were sacrificed by inhaling CO_2_. The tumor tissues were excised, then the volume and weight of the tumor tissues were tested. Western blot was performed to detected the expression of FAM134B and autophagy-related proteins in the tumor tissues.

### 2.12 Statistical analysis

All experiments were performed in triplicate. Statistical analysis was executed with GraphPad Prism 8.0 (GraphPad Software, USA). Data were presented as the mean ± standard deviation (SD). To ascertain group differences, Student’s t test was employed. For multifaceted comparison studies, One-Way Analysis of Variance (ANOVA) methodology was implemented. Correlation between FAM134B expression and clinicopathologic characteristics of patients was analyzed using Chi-square test. Statistically significant results were determined when *P* value < 0.05.

## 3 Results

### 3.1 FAM134B was highly expressed in breast cancer

**Figure 1:**
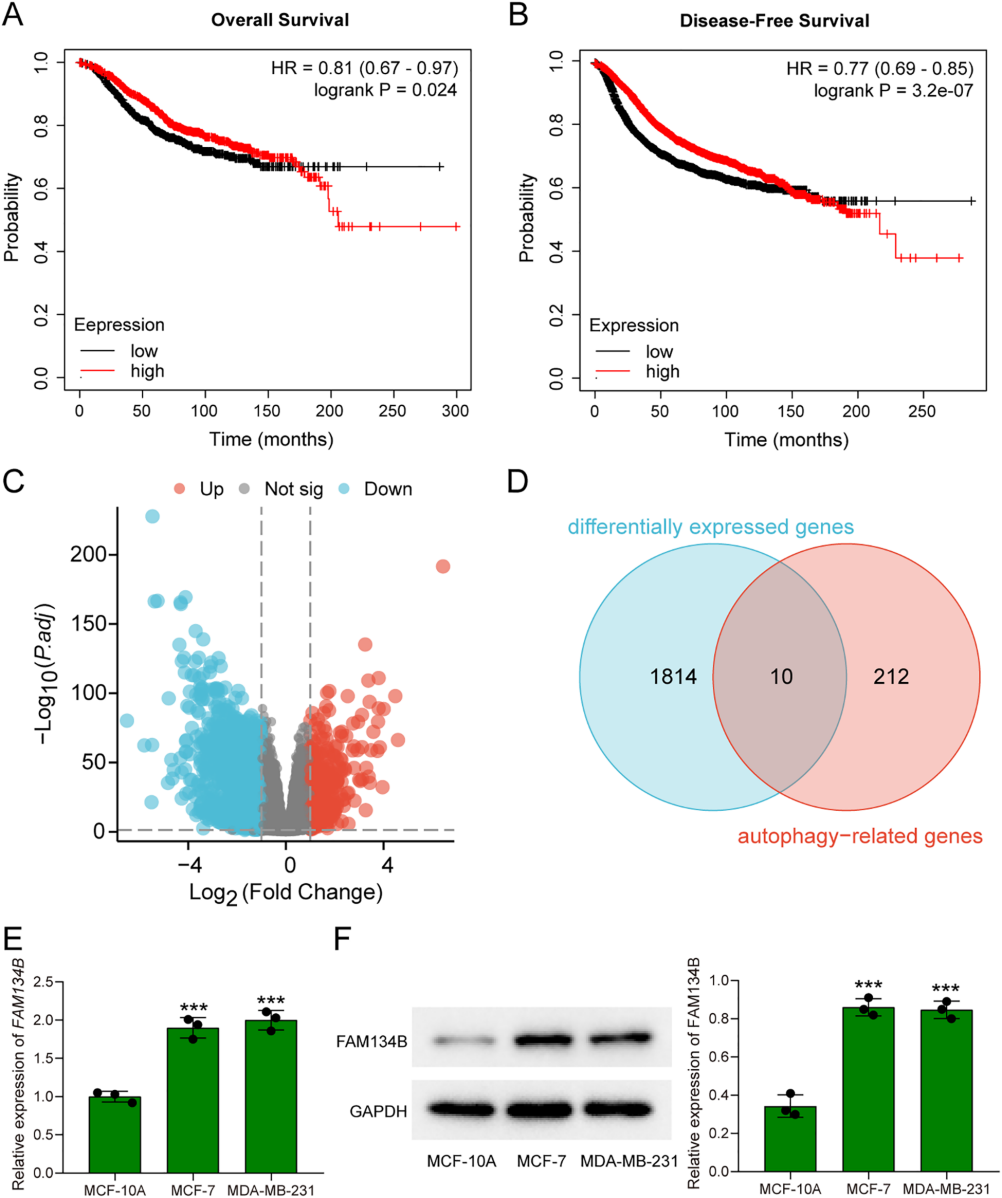
FAM134B was highly expressed in breast cancer. **(A)** The overall survival rates of breast cancer patients with high and low expression of *FAM134B*. **(B)** The disease-free survival rates of breast cancer patients with high and low expression of *FAM134B*. **(C)** Volcano plot of differential expressed genes. **(D)** Venn diagram of differential expressed genes and autophagy-related genes. **(E)** The expression of *FAM134B* mRNA was detected by qRT-PCR. **(F)** Western blot was used to evaluate the expression of FAM134B in cells. ***P<0.001 compared with MCF-10A cells.

In previous study, we have already discovered that FAM134B can promote the progression of hepatocellular carcinoma, in order to explore whether FAM134B also plays a role in breast cancer, a comprehensive analysis of its correlation with survival rates among breast cancer patients was initially conducted using the Kaplan-Meier Plotter database. Based on the TCGA dataset, breast cancer patients were divided into FAM134B high-expression and low-expression groups using the median expression level of FAM134B as the cutoff.The results indicated that patients with elevated levels of *FAM134B* exhibited reduced OS (*P* = 0.024, **Figure 1A**) and DFS (P = 3.2e^-07^, **Figure 1B**) compared to those expressing lower levels of *FAM134B*. Correlation analysis between FAM134B expression and clinicopathological characteristics was performed. Results demonstrated that FAM134B expression was significantly associated with patient age, Pathologic T stage, progesterone receptor (PR) status, estrogen receptor status, and human epidermal growth factor receptor 2 (HER2) status (**Table 2**). We also conducted an analysis of the differentially expressed genes between these two groups. For differential expression analysis, criteria of adjusted p.adjust)<0.05 and log_2_ fold change (log2FC)≥1 or ≤-1 were applied. A total of 1,824 differentially expressed mRNAs were identified, including 419 significantly upregulated and 1,405 significantly downregulated in the FAM134B high-expression group (**Figure 1C**). The differential expression results were visualized by a volcano plot (figure omitted). Subsequently, an intersection analysis was conducted between the 1,824 differentially expressed genes and autophagy-related genes (Human Autophagy Database, HADb, https://www.autophagy.lu/), and Venn diagram revealed 10 common genes (**Figure 1D**, **Table 3**). These 10 autophagy related genes showed significant differences between the FAM134B high-expression group and the FAM134B low-expression group, suggesting that FAM134B may be associated with autophagy in breast cancer. Moreover, investigations employing qRT-PCR on human normal breast epithelial cells (MCF-10A) and breast cancer cells (MCF-7 and MDA-MB-231), detected higher expression of *FAM134B* mRNA in breast cancer cells (*P*<0.001, **Figure 1E**). Western blotting further indicated an enhanced level of FAM134B protein expressed in breast cancer cells compared to normal breast epithelium cells (*P*<0.001, **Figure 1F**). These findings suggested that FAM134B might contribute to the progression of breast cancer.

**Table 2:** Correlation between FAM134B expression and clinicopathologic characteristics of breast cancer.

Characteristics	Low expression of FAM13B (543)	High expression of FAM134B(544)	P value
Age, n (%)			**<0.001**
<= 60	338 (31.1%)	265 (24.4%)	
> 60	205 (18.9%)	279 (25.7%)	
Pathologic T stage, n (%)			**0.036**
T1&T2	468 (43.2%)	441 (40.7%)	
T3&T4	75 (6.9%)	100 (9.2%)	
Pathologic N stage, n (%)			0.158
N0	270 (25.3%)	246 (23%)	
N1&N2&N3	265 (24.8%)	287 (26.9%)	
Pathologic M stage, n (%)			0.647
M0	454 (49.1%)	451 (48.8%)	
M1	9 (1%)	11 (1.2%)	
Pathologic stage, n (%)			0.359
Stage I	90 (8.5%)	92 (8.7%)	
Stage II	324 (30.5%)	295 (27.8%)	
Stage III	112 (10.5%)	132 (12.4%)	
Stage IV	8 (0.8%)	10 (0.9%)	
PR status, n (%)			**<0.001**
Negative	265 (25.6%)	77 (7.4%)	
Positive	258 (25%)	434 (42%)	
Estrogen Receptor status, n (%)			**<0.001**
Negative	219 (21.1%)	21 (2%)	
Positive	306 (29.5%)	491 (47.3%)	
HER2 status, n (%)			**0.005**
Negative	265 (37%)	295 (41.1%)	
Positive	94 (13.1%)	63 (8.8%)	

PR, Progesterone Receptor; HER2, Human Epidermal Growth Factor Receptor 2.

The bold values means statistic difference.

**Table 3:** The common genes of differential genes and autophagy-related genes.

Gene name	log_2_FoldChange	P value	P adj
CX3CL1	-1.222658152	2.58784E-48	1.38725E-46
IFNG	-1.198454763	2.36842E-19	2.01232E-18
NKX2-3	-1.413392856	1.12897E-08	4.04311E-08
ERBB2	-1.141069965	2.03911E-31	4.0016E-30
EGFR	-1.591966655	1.27102E-64	1.69521E-62
CDKN2A	-1.843002324	3.54323E-80	1.02494E-77
ITPR1	1.025461	1.90596E-48	1.0279E-46
NRG2	-1.305244402	2.08833E-34	5.00154E-33
TMEM74	-1.646903772	5.14488E-49	2.86845E-47
BCL2	1.120074038	6.93986E-54	5.04323E-52

### 3.2 FAM134B knockdown suppressed proliferation of breast cancer cells

**Figure 2:**
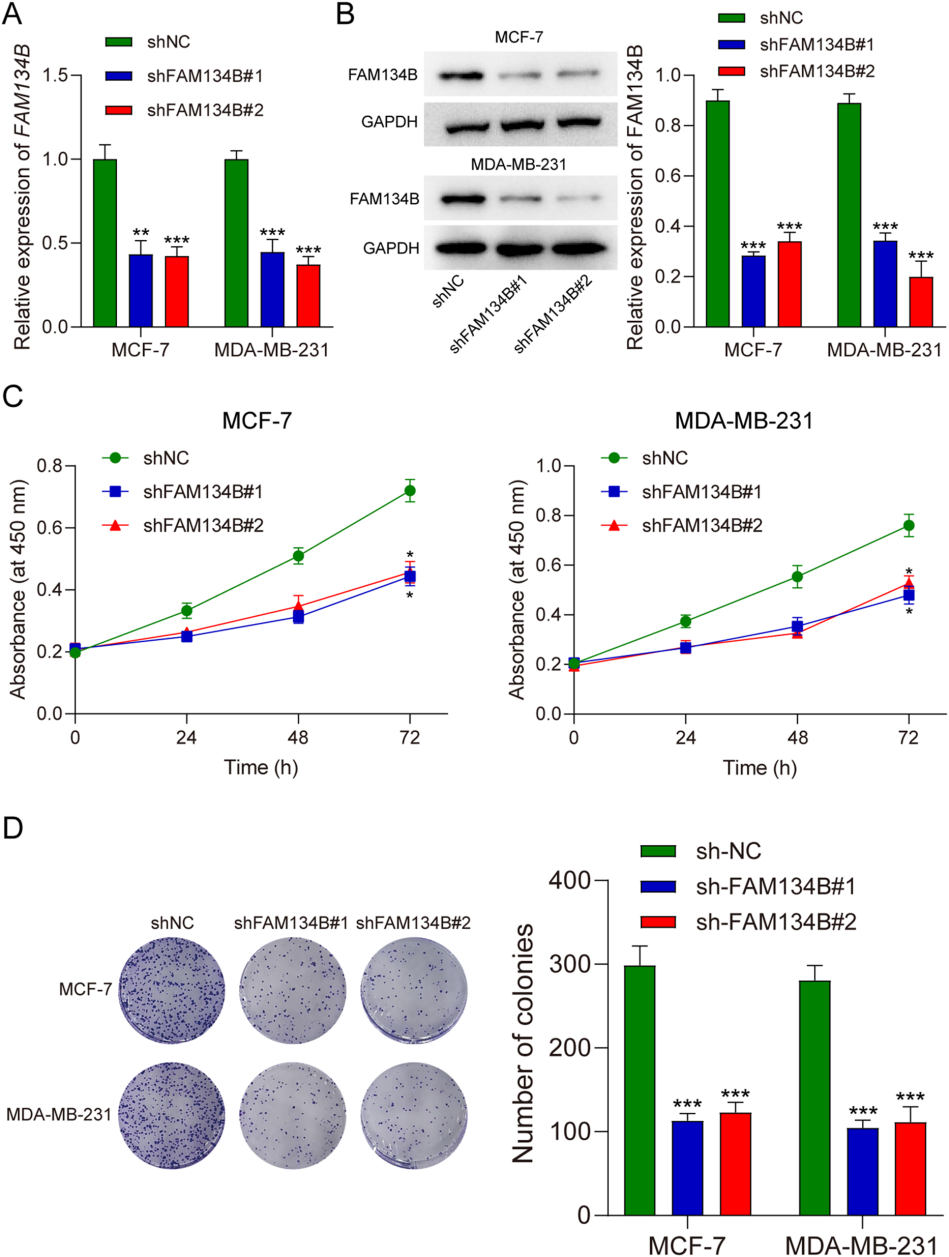
FAM134B knockdown suppressed proliferation of breast cancer cells. **(A)** The level of *FAM134B* mRNA was accessed by qRT-PCR after transfection. **(B)** The expression of FAM134B protein was detected using western blot after transfection. **(C)** CCK-8 assay was performed to calculate cell viability. **(D)** The proliferation ability of breast cancer cells was tested through clone formation assay. *P<0.05, **P<0.01, ***P<0.001 compared with shNC group.

qRT-PCR and western blotting were used to verify transfection efficiency. The qRT-PCR outcome indicated a reduction in *FAM134B* expression levels within the cells post transfection with shFAM134B (*P*<0.01, **Figure 2A**), and the results of western blot showed similar results on the level of FAM134B protein (*P*<0.001, **Figure 2B**), demonstrating successful transfection. The results of the CCK-8 assay indicated that the proliferation ability of breast cancer cells decreased after silencing FAM134B (*P*<0.05, **Figure 2C**). Colony formation assays further demonstrated that the number of colonies in the shFAM134B group was significantly lower than that in the shNC group (**Figure 2D**). These results indicated that FAM134B knockdown inhibited breast cancer cell proliferation.

### 3.3 FAM134Bsilencing promoted apoptosis and autophagy of breast cancer cells

**Figure 3:**
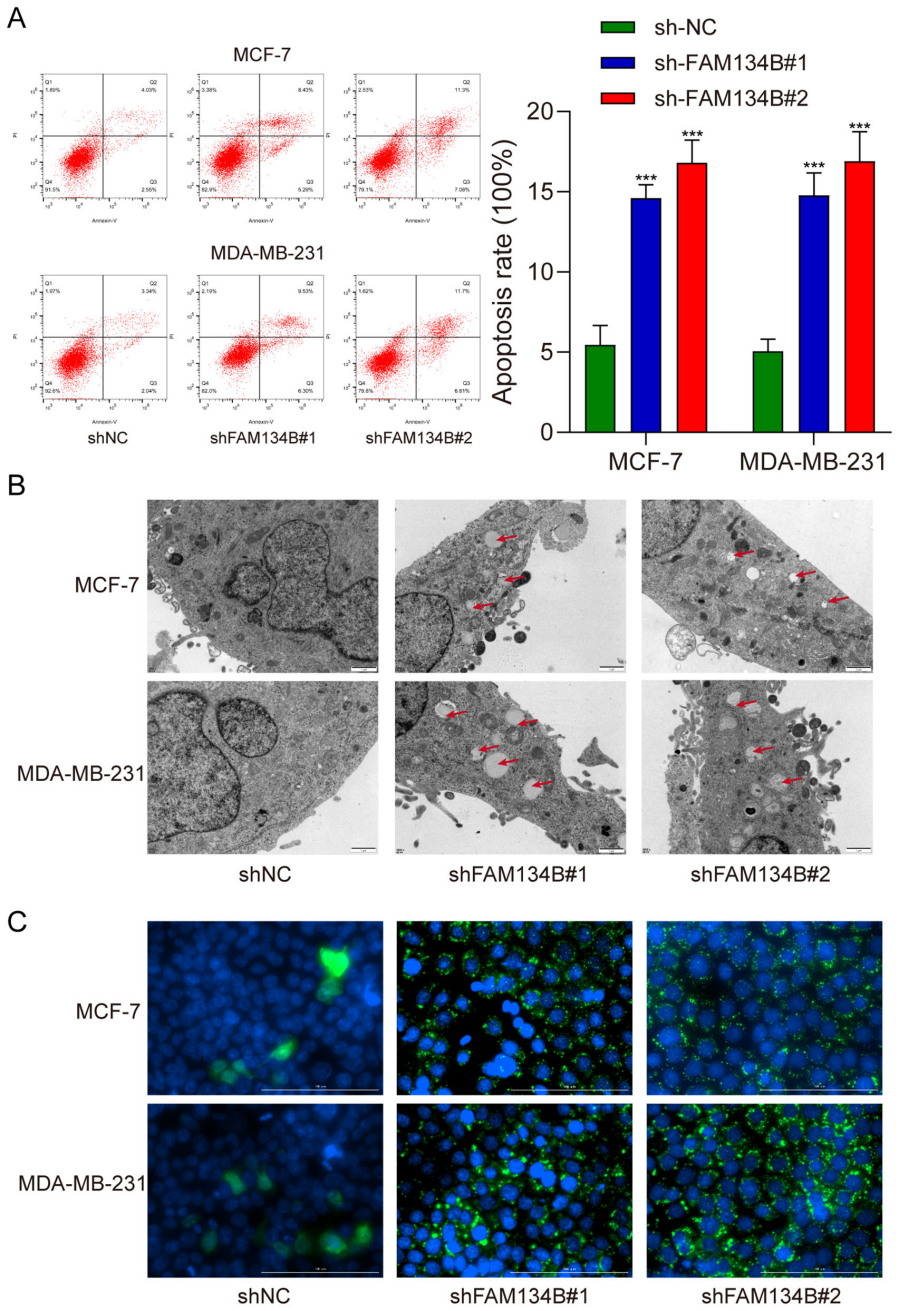
Silencing FAM134B promoted apoptosis and autophagy in breast cancer cells. **(A)** Flow cytometry was used to calculate the apoptosis rate of breast cancer cells. **(B)** Transmission electron microscopy was used to observe the formation of autophagosome in breast cancer cells. **(C)** Immunofluorescence microscopy assay was performed to explore the expression of LC3 in breast cancer cells. The green fluorescence indicates LC3 protein. ***P<0.001 compared with shNC group.

Subsequently, we examined the apoptosis rate of breast cancer cells post transfection by flow cytometry assay. The results indicated a significant increase in the apoptosis rate of breast cancer cells following silence of FAM134B (*P*<0.001, **Figure 3A**). Transmission electron microscopy revealed an increase in the number and size of autophagosomes in shFAM134B-transfected cells compared to shNC-transfected cells (**Figure 3B**). Furthermore, the experimental findings from immunofluorescence demonstrated a higher content of LC3 protein in the shFAM134B group compared to the control group (**Figure 3C**). These results suggest that downregulation of FAM134B induced apoptosis and autophagy in breast cancer cells.

### 3.4 shFAM134B promoted the expression of ER stress related proteins in breast cancer cells

**Figure 4:**
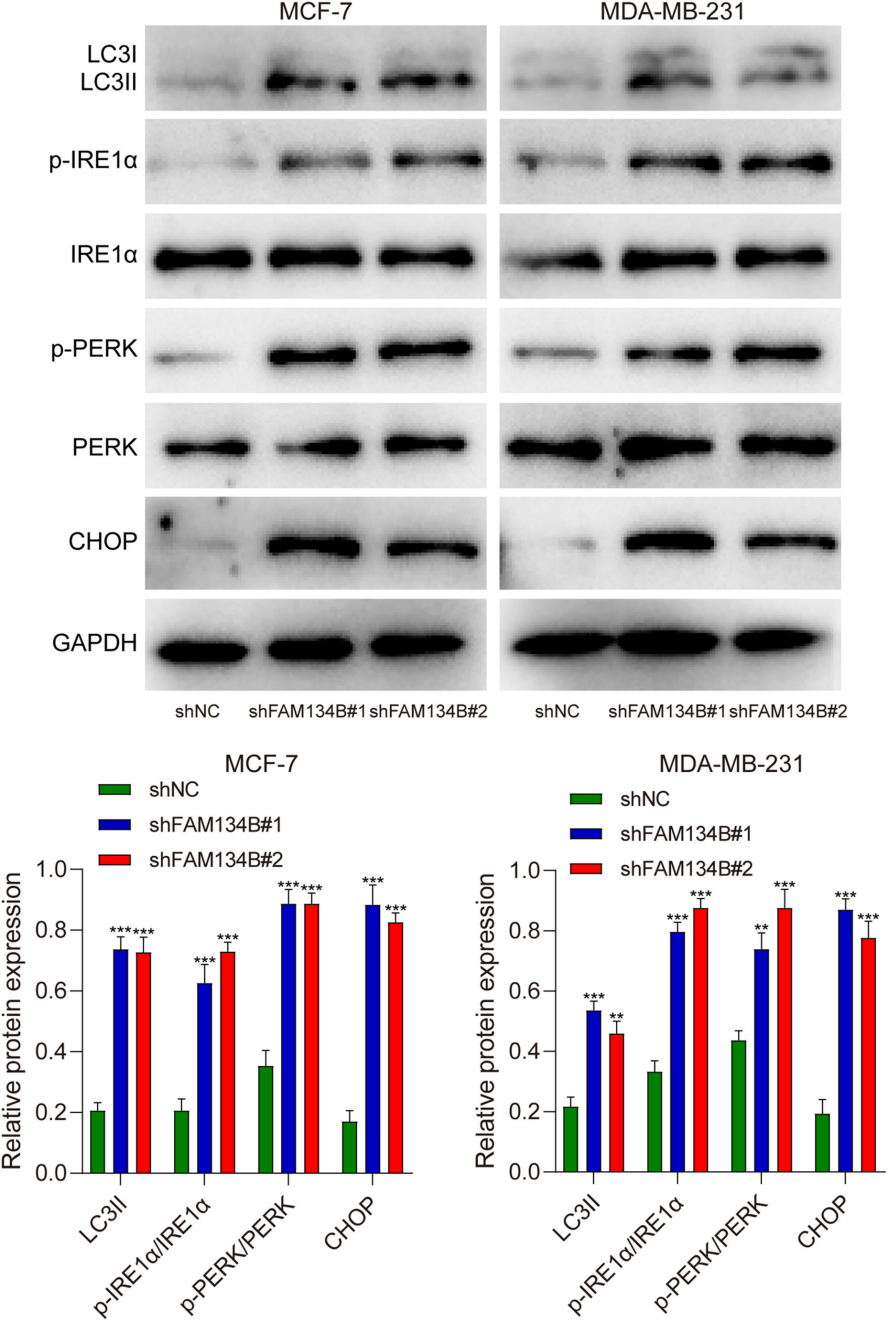
The effects of shFAM134B on the expression of autophagy and ER stress-related proteins. The expression of autophagy-related (LC3I/II) and ER stress-related proteins (IRE1α, p-IRE1α, PERK, p-PERK, CHOP) was detected using western blot. **P<0.01, ***P<0.001 compared with shNC group.

To further investigate the mechanism of the influence of FAM134B on breast cancer, we examined the expression levels of LC3I, LC3II, IRE1α, p-IRE1α, PERK, p-PERK, and CHOP as markers for autophagy and ER stress in breast cancer cells through western blot. The results revealed a significant increase in the expression of LC3II protein after downregulating FAM134B (*P*<0.01). Furthermore, the expression levels of p-IRE1α, p-PERK, and CHOP proteins in the shFAM134B group were significantly elevated compared to the control group (*P*<0.01, **Figure 4**). The findings suggested that downregulation of FAM134B stimulated autophagy and induce ER stress in breast cancer cells.

### 3.5 FAM134B knockdown inhibited the progression of breast cancer *in vivo*

**Figure 5:**
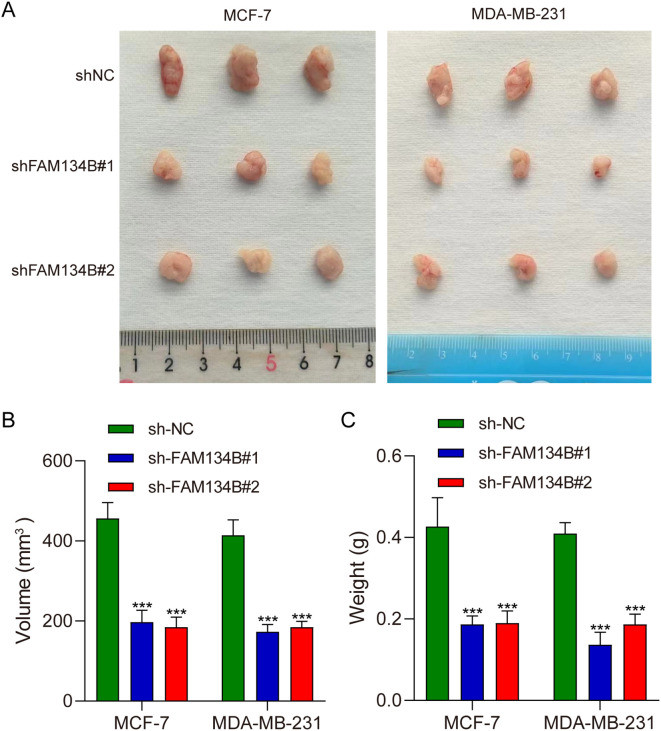
FAM134B knockdown inhibited the progression of breast cancer *in vivo*. **(A)** Representative images of tumors from nude mice injected with shFAM134B- or shNC-transfected MCF-7 and MDA-MB-231 cells (21 days post-injection). **(B)** The volume of the tumors. **(C)** The weight of the tumors. ***P<0.001 compared with shNC group.

**Figure 6:**
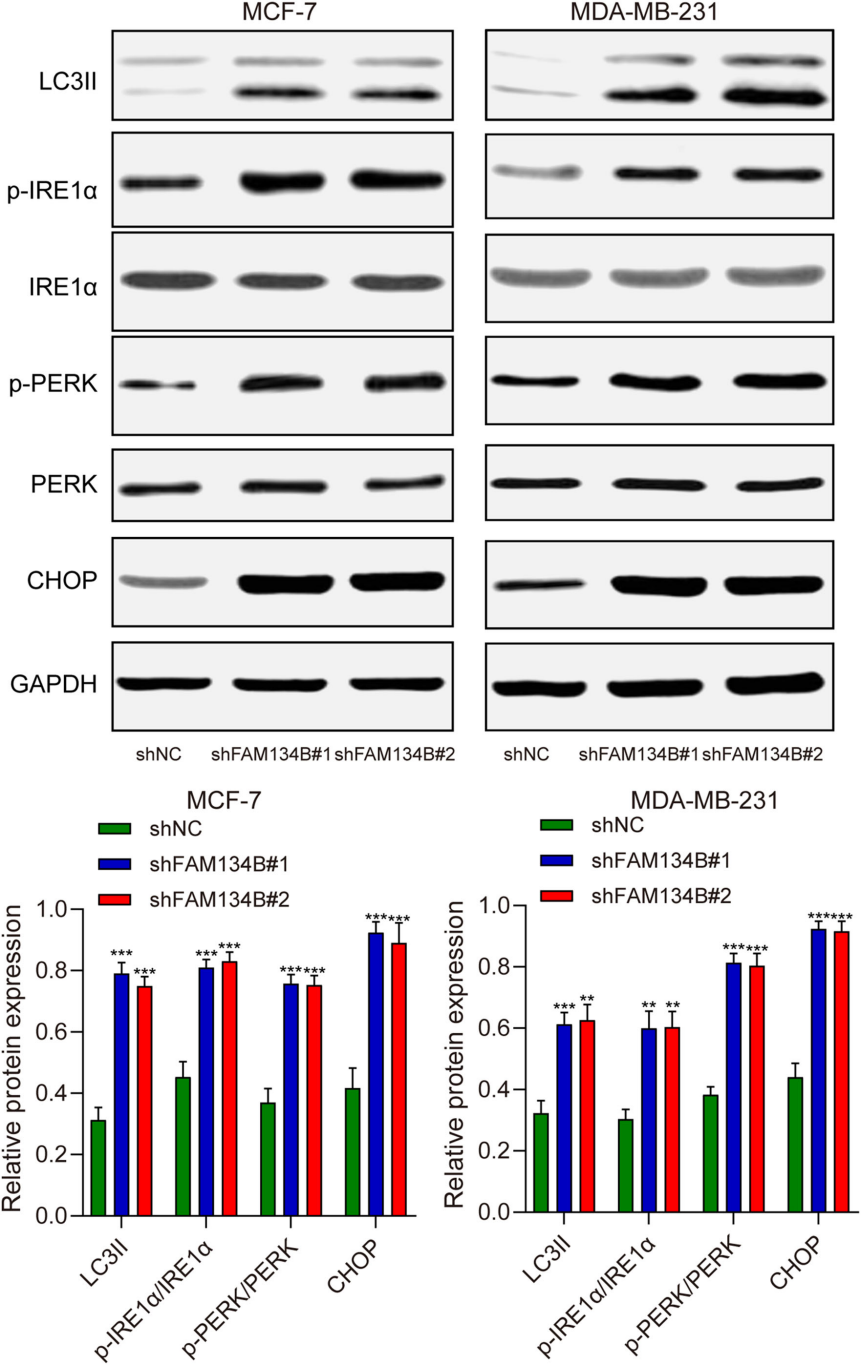
The effects of shFAM134B on the expression of autophagy and ER stress-related proteins *in vivo*. The expression of autophagy-related (LC3I/II) and ER stress-related proteins (IRE1α, p-IRE1α, PERK, p-PERK, CHOP) was detected using western blot. **P<0.01, ***P<0.001 compared with shNC group.

Tumor xenograft experiment was performed to verify the effect of FAM134B *in vivo*. The mice were euthanized 21 days post-injected with tumor cells, and the tumor tissues were isolated (**Figure 5A**). The experimental results indicated that the volume (*P*<0.001, **Figure 5B**) and weight (*P*<0.001, **Figure 5C**) of the tumor tissues were lower in shFAM134B than those in shNC group. In addition, the expression levels of LC3I, LC3II, IRE1α, p-IRE1α, PERK, p-PERK, and CHOP in tumor tissues were detected through western blot. The results revealed an increase in the expression of LC3II protein after downregulating FAM134B (*P*<0.01). Furthermore, the levels of p-IRE1α, p-PERK, and CHOP proteins in the shFAM134B group were significantly risen compared to the shNC group (*P*<0.01, **Figure 6**). These results indicated that FAM134B knockdown suppressed the growth of breast cancer not only *in vitro* but also *in vivo*.

## 4 Discussion

Breast cancer is the primary contributor to cancer‐induced mortality, and is also the most commonly identified malignancy among women globally (15). In current study, we found that breast cancer patients with high FAM134B expression had poorer OS and DFS than those with low FAM134B expression. Silence of FAM134B suppressed breast cancer cells proliferation, increased apoptosis, induced autophagy, and activated the expression of ER stress-related proteins. Our findings indicate that FAM134B act as an oncogene in breast cancer and may serve as a novel biomarker for the diagnosis of breast cancer.

FAM134B is a transmembrane protein localized to the ER membrane. Under physiological conditions, it participates in biological processes such as cell apoptosis and stress responses (16). Previous researches have demonstrated the pivotal role of FAM134B in the progression of neoplasms. For example, FAM134B promotes the development of esophageal squamous cell carcinoma *in vitro*, and patients with low expression of FAM134B exhibit better prognosis (11). In our previous study, we also found that FAM134B induces the progression and epithelial‐to‐mesenchymal transition of hepatocellular carcinoma by Akt signaling pathway (14). Consistent with these findings, the present study demonstrates that FAM134B functions as an oncogene in breast cancer.]

ER stress has been reported to play a vital role in cancer development (17, 18). The ER is responsible for protein secretion and appropriate protein folding, which are essential for maintaining protein homeostasis (19). Mild ER stress helps cancer cells and immune cells adapt to the tumor microenvironment, thereby promoting cancer cell proliferation, metastasis, and drug resistance. Conversely, severe ER stress can trigger immunogenic cell death and anti-tumor immunity (20). Some investigations have indicated that rigorous ER stress mediated through intricate signal transduction pathways stimulates autophagy, and in addition, the autophagy serves to arbitrate the survival of cells concurrently with ER stress (21). As a regulator of autophagy-dependent lysosomal degradation of ER proteins, FAM134B plays a crucial role in preventing the accumulation of harmful by-products of protein biosynthesis (22, 23). Preserving normal ER architecture and regulating ER homeostasis are the primary physiological functions of FAM134B (24, 25). Recent research indicated that FAM134B inhibits hepatocellular carcinoma cell autophagy, and promotes the development of liver cancer though inhibiting the expression of ER stress-related proteins (26). In addition, excessive ER-phagy induced by FAM134B culminates in ER stress, the unfolded protein response, and cell autophagy in cervical cancer cells (27). The possible mechanism by which FAM134B regulates ER stress and autophagy is that misfolded procollagen binds to calnexin, forming a transient yet relatively stable complex. This calnexin-misfolded procollagen complex further interacts with the reticulon homology domain (RHD) of FAM134B, leading to the formation of a FAM134B-calnexin complex. Concurrently, FAM134B utilizes its RHD to induce ER membrane curvature. FAM134B additionally employs its LC3-interacting region (LIR) to recruit LC3, thereby facilitating the formation of autophagic vesicles. Ultimately, both misfolded procollagen and calnexin are sequestered within these autophagic vesicles and subsequently transported to lysosomes for degradation (22, 24). In the present study, we also discovered that inhibition of FAM134B activated the expression of ER stress related protein p-IRE1α, PERK as well as CHOP, and induced autophagy. These results indicated that FAM134B might regulate the progression of breast cancer by modulating ER stress-induced autophagy.

Currently, there are several limitations in our research. For example, due to the ethical restrictions, the analysis of FAM134B’s clinical value relies exclusively on public databases, without validation using independent clinical samples from local breast cancer cohorts. On the other hand, although we confirmed that FAM134B modulates breast cancer progression through ER stress and autophagy, the specific downstream pathways involved and its crosstalk with other key signaling pathways remain unclear. In future studies, we aim to overcome these constraints by validating with additional clinical samples and conducting in-depth investigations into the underlying molecular mechanisms, thereby enhancing the generalizability of our findings and their clinical applicability.

## 5 Conclusion

In summary, this investigation demonstrates that FAM134B expression correlates with poor prognosis in breast cancer patients. Silencing of FAM134B inhibits breast cancer progression by suppressing cell proliferation, stimulating apoptosis, activating autophagy and triggering ER stress. Our research identifies FAM134B as a potential biomarker for diagnostics of breast cancer, and provides a therapeutic target for breast cancer.

## Data Availability

The original contributions presented in the study are included in the article/supplementary material. Further inquiries can be directed to the corresponding authors.

## References

[1] Ficarra S Thomas E Bianco A Gentile A Thaller P Grassadonio F . Impact of exercise interventions on physical fitness in breast cancer patients and survivors: a systematic review. Breast Cancer. (2022) 29:402–18. doi: 10.1007/s12282-022-01347-z, PMID: 35278203 PMC9021138

[2] Siegel RL Giaquinto AN Jemal A . Cancer statistic. CA Cancer J Clin. (2024) 74:12–49. doi: 10.3322/caac.21820, PMID: 38230766

[3] Sung H Ferlay J Siegel RL Laversanne M Soerjomataram I Jemal A . Global cancer statistics 2020: GLOBOCAN estimates of incidence and mortality worldwide for 36 cancers in 185 countries. CA Cancer J Clin. (2021) 71:209–49. doi: 10.3322/caac.21660, PMID: 33538338

[4] Rozenberg S Di Pietrantonio V Vandromme J Gilles C . Menopausal hormone therapy and breast cancer risk. Best Pract Res Clin Endocrinol Metab. (2021) 35:101577. doi: 10.1016/j.beem.2021.101577, PMID: 34535397

[5] Oakes SA . Endoplasmic reticulum stress signaling in cancer cells. Am J Pathol. (2020) 190:934–46. doi: 10.1016/j.ajpath.2020.01.010, PMID: 32112719 PMC7237829

[6] Ajoolabady A Kaplowitz N Lebeaupin C Kroemer G Kaufman RJ Malhi H . Endoplasmic reticulum stress in liver diseases. Hepatology. (2023) 77:619–39. doi: 10.1002/hep.32562, PMID: 35524448 PMC9637239

[7] Molinari M . ER-phagy: eating the factory. Mol Cell. (2020) 78:811–3. doi: 10.1016/j.molcel.2020.05.002, PMID: 32502421

[8] Smith MD Harley ME Kemp AJ Wills J Lee M Arends M . CCPG1 is a non-canonical autophagy cargo receptor essential for ER-phagy and pancreatic ER proteostasis. Dev Cell. (2018) 44:217–232 e11. doi: 10.1016/j.devcel.2017.11.024, PMID: 29290589 PMC5791736

[9] Oakes SA Papa FR . The role of endoplasmic reticulum stress in human pathology. Annu Rev Pathol. (2015) 10:173–94. doi: 10.1146/annurev-pathol-012513-104649, PMID: 25387057 PMC5568783

[10] Ingle J Tirkey A Pandey S Basu S . Small-molecule endoplasmic reticulum stress inducer triggers apoptosis in cancer cells. ChemMedChem. (2023) 18:e202300433. doi: 10.1002/cmdc.202300433, PMID: 37964696

[11] Islam F Gopalan V Law S Tang JC Lam AK . FAM134B promotes esophageal squamous cell carcinoma *in vitro* and its correlations with clinicopathologic features. Hum Pathol. (2019) 87:1–10. doi: 10.1016/j.humpath.2018.11.033, PMID: 30794892

[12] Haque MH Gopalan V Chan KW Shiddiky MJ Smith RA Lam AK . Identification of novel FAM134B (JK1) mutations in oesophageal squamous cell carcinoma. Sci Rep. (2016) 6:29173. doi: 10.1038/srep29173, PMID: 27373372 PMC4931577

[13] Khaminets A Heinrich T Mari M Grumati P Huebner AK Akutsu M . Regulation of endoplasmic reticulum turnover by selective autophagy. Nature. (2015) 522:354–8. doi: 10.1038/nature14498, PMID: 26040720

[14] Zhang ZQ Chen J Huang WQ Ning D Liu QM Wang C . FAM134B induces tumorigenesis and epithelial-to-mesenchymal transition via Akt signaling in hepatocellular carcinoma. Mol Oncol. (2019) 13:792–810. doi: 10.1002/1878-0261.12429, PMID: 30556279 PMC6441892

[15] Lei S Zheng R Zhang S Wang S Chen R Sun K . Global patterns of breast cancer incidence and mortality: A population-based cancer registry data analysis from 2000 to 2020. Cancer Commun (Lond). (2021) 41:1183–94. doi: 10.1002/cac2.12207, PMID: 34399040 PMC8626596

[16] Cinque L De Leonibus C Iavazzo M Krahmer N Intartaglia D Salierno FG . MiT/TFE factors control ER-phagy via transcriptional regulation of FAM134B. EMBO J. (2020) 39:e105696. doi: 10.15252/embj.2020105696, PMID: 32716134 PMC7459426

[17] Chen X Cubillos-Ruiz JR . Endoplasmic reticulum stress signals in the tumour and its microenvironment. Nat Rev Cancer. (2021) 21:71–88. doi: 10.1038/s41568-020-00312-2, PMID: 33214692 PMC7927882

[18] Kim C Kim B . Anti-cancer natural products and their bioactive compounds inducing ER stress-mediated apoptosis: A review. Nutrients. (2018) 10:1021. doi: 10.3390/nu10081021, PMID: 30081573 PMC6115829

[19] Sisinni L Pietrafesa M Lepore S Maddalena F Condelli V Esposito F . Endoplasmic reticulum stress and unfolded protein response in breast cancer: the balance between apoptosis and autophagy and its role in drug resistance. Int J Mol Sci. (2019) 20:857. doi: 10.3390/ijms20040857, PMID: 30781465 PMC6412864

[20] Xu D Liu Z Liang MX Fei YJ Zhang W Wu Y . Endoplasmic reticulum stress targeted therapy for breast cancer. Cell Commun Signal. (2022) 20:174. doi: 10.1186/s12964-022-00964-7, PMID: 36345017 PMC9639265

[21] Zhu J Tian S Li KT Chen Q Jiang Y Lin HD . Inhibition of breast cancer cell growth by methyl pyropheophenylchlorin photodynamic therapy is mediated though endoplasmic reticulum stress-induced autophagy. Vitro vivo Cancer Med. (2018) 7:1908–20. doi: 10.1002/cam4.1418, PMID: 29577663 PMC5943539

[22] Chen W Mao H Chen L Li L . The pivotal role of FAM134B in selective ER-phagy and diseases. Biochim Biophys Acta Mol Cell Res. (2022) 1869:119277. doi: 10.1016/j.bbamcr.2022.119277, PMID: 35477002

[23] Jiang X Wang X Ding X Du M Li B Weng X . FAM134B oligomerization drives endoplasmic reticulum membrane scission for ER-phagy. EMBO J. (2020) 39:e102608. doi: 10.15252/embj.2019102608, PMID: 31930741 PMC7049798

[24] Forrester A De Leonibus C Grumati P Fasana E Piemontese M Staiano L . A selective ER-phagy exerts procollagen quality control via a Calnexin-FAM134B complex. EMBO J. (2019) 38:e99847. doi: 10.15252/embj.201899847, PMID: 30559329 PMC6331724

[25] Siggel M Bhaskara RM Moesser MK Ikić DI Hummer G . FAM134B-RHD protein clustering drives spontaneous budding of asymmetric membranes. J Phys Chem Lett. (2021) 12:1926–31. doi: 10.1021/acs.jpclett.1c00031, PMID: 33591770 PMC8028312

[26] Wang H Liu L Gong H Li H . Upregulation of FAM134B inhibits endoplasmic reticulum stress-related degradation protein expression and promotes hepatocellular carcinogenesis. J Cell Mol Med. (2023) 28:e17964. doi: 10.1111/jcmm.17964, PMID: 37728036 PMC10902567

[27] Liao Y Duan B Zhang Y Zhang X Xia B . Excessive ER-phagy mediated by the autophagy receptor FAM134B results in ER stress, the unfolded protein response, and cell death in HeLa cells. J Biol Chem. (2019) 294:20009–23. doi: 10.1074/jbc.RA119.008709, PMID: 31748416 PMC6937584

